# Complete clinical regression of a BRAF V600E-mutant pediatric glioblastoma multiforme after BRAF inhibitor therapy

**DOI:** 10.1186/1471-2407-14-258

**Published:** 2014-04-12

**Authors:** Giles W Robinson, Brent A Orr, Amar Gajjar

**Affiliations:** 1 Division of Neuro-Oncology, Department of Oncology, St Jude Children’s Research Hospital, 262 Danny Thomas Place, Memphis, TN 38105, USA; 2 Department of Pathology, St Jude Children’s Research Hospital, Memphis, TN, USA

**Keywords:** High-grade glioma, Glioblastoma multiforme, *BRAF* mutations, *V600E*, Pediatric brain tumor, *BRAF* inhibitors

## Abstract

**Background:**

Standard therapies for high grade glioma have failed to substantially improve survival and are associated with significant morbidity. At relapse, high grade gliomas, such as glioblastoma multiforme, are refractory to therapy and universally fatal. *BRAF V600E*-mutations have been described in a modest 6% to 7% of primary central nervous system (CNS) tumors, but with increased prevalence in the pediatric population and in certain brain tumor subtypes. The use of *BRAF* inhibitors have transformed melanoma therapy however their use in brain tumors remains unproven.

**Case presentation:**

We describe the pediatric case of a now 12 year old Caucasian male who originally presented at age 9 with a right fronto-parietal glioblastoma multiforme that recurred 2 ½ years from diagnosis. Molecular analysis of the primary tumor revealed a *BRAF V600E* mutation and the patient was placed on the *BRAF* inhibitor vemurafenib. A complete response was observed after 4 months of therapy and remains sustained at 6 months.

**Conclusion:**

This is the first report of a complete response of relapsed glioblastoma multiforme to targeted *BRAF* inhibitor therapy. While not a predominant mutation in glioblastoma multiforme, the increased prevalence of *BRAF V600* mutations in pediatric CNS tumors and certain subtypes marks a population to whom this therapy could be applied. Response to this therapy suggests that BRAF inhibitors can affect primary CNS lesions when a documented and targetable mutation is present.

## Background

The highest incidence of CNS tumors that harbor *BRAF V600E*-mutations occurs in pediatric patients [1]. In particular, a relatively high frequency of these mutations has been identified in pediatric pilocytic astrocytomas, pleomorphic xanthoastrocytomas, malignant astrocytomas, gangliogliomas, and the epithelioid subtype of glioblastoma multiforme [1-4]. Although *BRAF* inhibitors extend survival and improve the quality of life in patients with *BRAF V600E*-mutated melanoma [5,6], variable responses to *BRAF* inhibitors have been described in different tumor types [7]. Additionally, although melanoma that has metastasized to the CNS responds to *BRAF* inhibitors [6], these metastases do not have an intact blood–brain barrier [8], which frequently blocks an agent’s ability to reach CNS tumors at exposures and concentrations necessary to achieve the desired pharmacologic effect. Therefore, it is unclear whether *BRAF* inhibition can clinically affect a primary CNS lesion as it does a secondary one. Here we describe the first known case of complete response in a *BRAF V600E*-mutated high-grade glioma to vemurafenib (*BRAF* inhibitor) therapy.

### Case presentation

**Figure 1:**
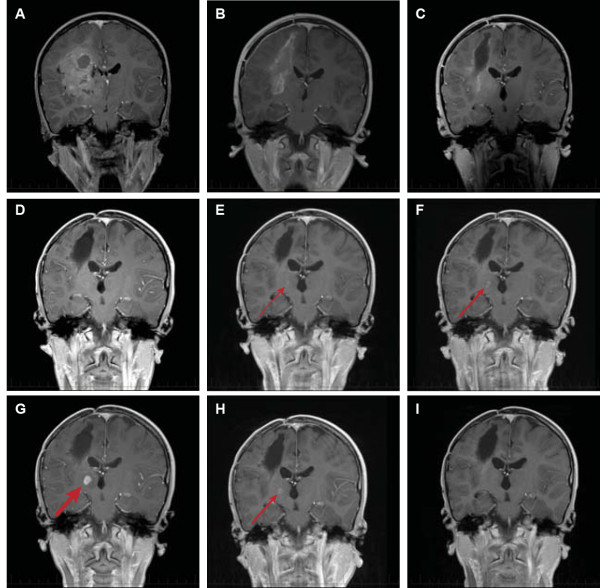
**Chronological changes on magnetic resonance imaging (MRI) document the tumor recurrence and response.** Coronal MRI T1-weighted images with gadolinium-based contrast were taken at the following times: **(A)** diagnosis, **(B)** post-operatively, **(C)** after completion of radiation therapy, **(D)** while receiving adjuvant chemotherapy, **(E)** at completion of therapy, **(F)** 4 months after completion of therapy, **(G)** upon start of vemurafenib therapy at relapse, **(H)** after 2 months of vemurafenib therapy, and **(I)** after 4 months of vemurafenib therapy.

**Figure 2:**
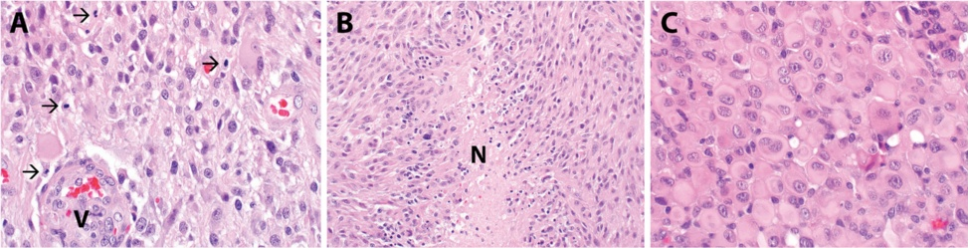
**The diagnosis of glioblastoma (WHO grade IV) was rendered on histopathologic review.** Histopathologic evaluation revealed a hypercellular astrocytic neoplasm which infiltrated the surrounding brain parenchyma. Mitotic activity (arrows) was abundant and microvascular proliferation (designated V) was present **(A)**. Necrosis was encountered in the specimen, including pseudo-palisading necrosis (designated N) **(B)**. While not a dominant appearance, focally the tumor had features of epithelioid glioblastoma **(C)**.

A 9-year-old patient presented with a one-week onset of progressive left-sided weakness. His symptoms were first noted by his father when the boy had difficulty extending the fingers on his left hand to catch an American football. Within a few days, a left leg limp and the beginnings of a left-sided facial droop had developed. Magnetic resonance imaging (MRI) revealed a large (7 cm × 5 cm × 5 cm), spherical heterogeneously enhancing, mixed cystic and solid mass in the right fronto-parietal region, with extension into the internal capsule, thalamus, and basal ganglia (Figure 1A). The solid tumor elements demonstrated restricted diffusion suggestive of high-grade tumor activity. A stereotactic right fronto-parietal craniotomy was performed, and the vast majority of the tumor was successfully removed (Figure 1B); however, the most medial structures of the right internal capsule and thalamus were spared an aggressive resection to preserve the patient’s neurologic function. Upon histopathologic review, the tumor consisted of a diffusely infiltrating glial neoplasm. The hypercellular tumor demonstrated mitotic activity, vascular proliferation, and palisading necrosis (Figure 2A and B), fulfilling criteria for glioblastoma (WHO grade IV). While not the dominant morphology, focally the tumor demonstrated features of the epithelioid variant of glioblastoma (Figure 2C).

Post-operative MRI scans showed residual tumor in the right thalamus, consistent with the operative description of remnants of tumor in this location. After recovering from surgery, the patient was treated with a best clinical management plan. Focal radiation of 59.4 Gy to the tumor bed was administered in combination with vorinostat (230 mg/m^2^/dose 5 days/week) therapy as a radiosensitizer over a 6-week period. After a 4 week break, he received combination chemotherapy with bevacizumab (10 mg/kg/dose every 2 weeks), topotecan (0.8 mg/m^2^/dose days 1–10), and vorinostat (180 mg/m^2^/dose days 1–14) administered over 28-day cycles. Regular MRI scans of the patient showed no evidence of disease progression while he was on therapy (Figure 1C-E), and what had previously been reported on radiology reports as residual disease in the thalamus was reported as probable enhancing gliosis with suspected regional mineralization. After a total of 24 months of therapy, he was taken off therapy and monitored with serial brain MRI on a tri-monthly basis.

Four months after stopping therapy, an area of new enhancement became apparent (Figure 1F). This focus was deep in the patient’s right thalamus and more medial to where the original residual disease was suspected to be. This focus was of concern because it arose within a region that was always closely monitored due to the presence of T2 prolongation. But the focus was initially small and thought to represent a nonspecific change within a heavily treated region. Subsequent scans, however, showed this focus to be enlarging (Figure 1F-G) with increased perfusion, consistent with a recurrent and progressive tumor. By 8 months from the end of therapy, this mass had a 1-cm diameter (Figure 1G), and the patient and family were informed that this was almost certainly a recurrent tumor.

**Figure 3:**
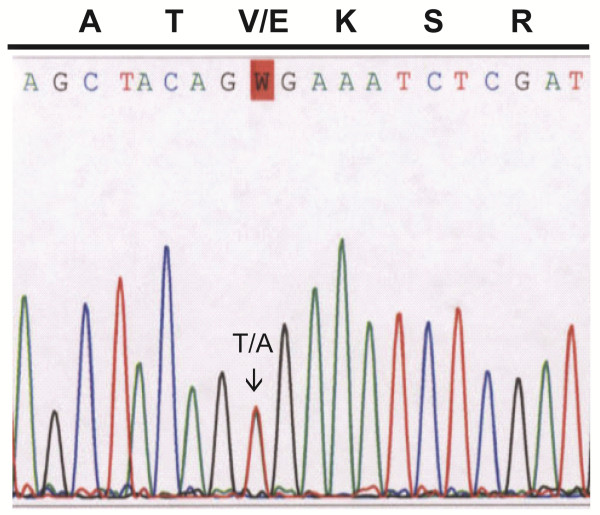
Electropherogram derived from patient’s tumor sample showing a point mutation at codon 600 (GTG to GAG) resulting in a Valine (V) to Glutamic acid (E) substitution.

Given the absence of symptoms in the patient, the infiltrative nature of the disease, and the location of the tumor focus deep in the thalamus, an attempt at surgical resection was judged to be a poor option with a high chance of morbidity and almost no chance of safely removing all microscopic disease. Similarly, the risk of biopsy did not outweigh the benefits of a histologic confirmation of an already highly malignant tumor. Re-initiation of the prior chemotherapy regimen was considered but not felt to be indicated because, in retrospect, the enhancing lesion was found to be present in a punctate form on the patient’s imaging just prior to stopping therapy (Figure 1E). Therefore, the pathology of the original tumor was again reviewed with this recurrence and additional molecular characterization of the tumor was performed. Based on the focal features of epithelioid glioblastoma multiforme, a variant previously reported to have a high proportion of *BRAF* abnormalities [2], *BRAF V600E* testing was performed on material extracted from the paraffin embedded tissue. By PCR amplification and subsequent sequencing, a *BRAF V600E* mutation was detected in the patient sample (Figure 3).

Vemurafenib, a *BRAF* inhibitor recently approved by the United States Food and Drug Administration for therapy of melanoma with a V600E mutation, was initiated as an off-label use. The child’s therapy was started at 720 mg by mouth twice daily for 28 days (about 600 mg/m^2^ per dose), which approximates the recommended adult dose of 920 mg by mouth twice daily. Two months from initiation of therapy, a follow-up MRI showed a partial response (Figure 1H). Four months from initiation of therapy, the recurrent tumor was no longer detectable by MRI (Figure 1I) and this effect continued through a six month MRI evaluation.

Therapy was initially halted after the first 5 consecutive days because a severe diffuse erythematous palpable follicular rash developed. When this rash almost completely resolved after 8 days off the medication, the drug was resumed at the same dose. The rash returned in a milder form and remained stable except for occasional flares in sun- and wind-exposed areas. Additional adverse events included partial alopecia, madarosis, and change in hair texture. Serial dermatologic exams revealed no evidence of dysplastic or neoplastic skin lesions, and family members were encouraged to adhere to strict precautions in the sun. Serial EKGs showed no prolongation of the corrected QT interval, and serial eye exams showed no ocular effects of the medication. The patient is on his seventh cycle of therapy under stringent observation.

## Conclusions

A complete response of a CNS tumor to *BRAF* inhibitor therapy underscores the need to fully investigate these targeted drugs in patients with CNS tumors which harbor *BRAF* mutations. While interest in utilizing these drugs in the CNS population has been rising, there has been little data to suggest that these drugs will be effective in these circumstances. This case suggests that a drug of this class can penetrate a primary brain tumor and affect a primary CNS lesion harboring a *BRAF* mutation. A dramatic response of this nature, however, must be received with cautious optimism.

The experience of *BRAF* inhibition in other tumor types suggests that response is unlikely to be uniform across all CNS tumors, even in the presence of similar *V600* mutations. Already, a sampling of four adult patients with *BRAF V600E*-mutated pleomorphic xanthoastrocytomas treated with vemurafenib shows that the best documented response is a modest, partial response [9]. Two pediatric patients with *BRAF V600E* mutated gangliogliomas have now been reported to have a sustained partial response while another two patients, one ganglioglioma and the other a malignant astrocytoma, had a transient (< 2 month) and no response, respectively [10,11]. While our case demonstrates that aggressive high grade gliomas can respond, pathways of resistance may already exist within these tumors. Reports in colorectal cancer suggest BRAF-mutant tumors may escape inhibition by amplifying receptor tyrosine kinases, such as epidermal growth factor receptor (EGFR), and EGFR amplification and fusion are common alterations found in adult glioblastoma multiforme lesions [7,12]. Moreover, the response of CNS tumors with alternative *BRAF* abnormalities, such as alternate *V600* mutations or fusions, will also need to be investigated. Preclinical data suggest that *BRAF* fusions, which are widespread in pilocytic astrocytoma, may not be as responsive to *BRAF* inhibitors as *V600*-mutated tumors are [13].

Unanticipated side effects are bound to surface, and the susceptible and vulnerable pediatric population will predictably remain at high risk. For example, blocking *RAF* kinase has been shown to paradoxically upregulate its activator *RAS*, leading to the formation of skin neoplasias and to the progression of *RAS*-mutated malignancies [14-17]. Therefore, what a blockade of *RAF* signaling in the *MAPK* pathway may do to a young developing child over a lifetime will need to be carefully documented in clinical trials.

The melanoma experience suggests that resistance will surely emerge in CNS tumors responsive to this therapy [14,18]. Therefore, coadministration with other *MAPK* pathway inhibitors, such as *MEK* inhibitors, will need to be investigated to prevent resistance from *MAPK* pathway reactivation [19,20]. Also, coadministration with alternative survival pathway inhibitors, such as *PI3K* inhibitors and *VEGF* inhibitors, may need to be evaluated [20].

In conclusion, this case provides evidence that *BRAF* inhibition has important therapeutic potential in CNS tumors, including the most aggressive high grade gliomas. These and other targeted agents provide hope for the treatment of advanced and incurable tumors and may radically improve current therapy. Substantial work remains to be done before we understand when and how to best use this new class of drugs, however the identification of the potential responders through careful histologic and mutational analysis is critical. Even if the effect of this targeted therapy remains temporary, therapeutic goals could include extending survival and improving quality of life in patients with relapsed disease, improving the extent of surgical resection of a tumor, and increasing time to radiation in order to preserve a child’s neurocognitive development.

## Consent

Written informed consent was obtained from the patient’s parents for publication of this Case report and accompanying images. A copy of the written consent is available for review by the Editor-in-Chief of this journal.

## Competing interests

There are no competing interests in the report.

## Authors’ contributions

GWR drafted the manuscript. BAO carried out the pathology studies. AG and BAO provided critical important intellectual revisions to the manuscript. All authors read and approved the final manuscript.

## Pre-publication history

The pre-publication history for this paper can be accessed here:

http://www.biomedcentral.com/1471-2407/14/258/prepub
